# Syringic acid mitigates isoproterenol‐induced cardiac hypertrophy and fibrosis by downregulating Ereg

**DOI:** 10.1111/jcmm.17449

**Published:** 2022-06-19

**Authors:** Xiongyi Han, Liyan Bai, Hae Jin Kee, Myung Ho Jeong

**Affiliations:** ^1^ Heart Research Center of Chonnam National University Hospital Gwangju Korea; ^2^ Hypertension Heart Failure Research Center Chonnam National University Hospital Gwangju Korea; ^3^ Department of Cardiology Chonnam National University Medical School Gwangju Korea

**Keywords:** cardiac hypertrophy, epiregulin, fibrosis, syringic acid

## Abstract

Gallic acid has been reported to mitigate cardiac hypertrophy, fibrosis and arterial hypertension. The effects of syringic acid, a derivative of gallic acid, on cardiac hypertrophy and fibrosis have not been previously investigated. This study aimed to examine the effects of syringic acid on isoproterenol‐treated mice and cells. Syringic acid mitigated the isoproterenol‐induced upregulation of heart weight to bodyweight ratio, pathological cardiac remodelling and fibrosis in mice. Picrosirius red staining, quantitative real‐time polymerase chain reaction (qRT‐PCR) and Western blotting analyses revealed that syringic acid markedly downregulated collagen accumulation and fibrosis‐related factors, including Fn1. The results of RNA sequencing analysis of *Ereg* expression were verified using qRT‐PCR. Syringic acid or transfection with si‐*Ereg* mitigated the isoproterenol‐induced upregulation of Ereg, Myc and Ngfr. *Ereg* knockdown mitigated the isoproterenol‐induced upregulation of Nppb and Fn1 and enhancement of cell size. Mechanistically, syringic acid alleviated cardiac hypertrophy and fibrosis by downregulating Ereg. These results suggest that syringic acid is a potential therapeutic agent for cardiac hypertrophy and fibrosis.

## 1 INTRODUCTION

Cardiomyocytes, which exhibit postmitotic characteristics, do not divide but exhibit growth in response to various stresses. The hallmarks of cardiomyocyte hypertrophy involve the activation of foetal gene program, upregulation of protein synthesis and reorganization of sarcomeres.^1^ Cardiac hypertrophy is a hallmark feature of various cardiovascular diseases, including myocardial infarction, hypertension, heart failure and valvular diseases.^2^ Although short‐term cardiac hypertrophy can be beneficial, long‐term cardiac hypertrophy is detrimental and must be effectively managed. Genes that suppress cardiac hypertrophy, including *KLF4* and *INPP5F*, are potential therapeutic targets for cardiac hypertrophy.^3^, ^4^, ^5^

In addition to drugs and genes, natural substances produced in plants and foods can suppress cardiac hypertrophy. Syringic acid, a phenolic compound found in various fruits, including olives, pumpkin and grapes^6^ is reported to mitigate myocardial ischaemia–reperfusion injury and cardiotoxicity.^7^, ^8^ Additionally, syringic acid exerts protective effects on streptozotocin‐induced diabetic cardiomyopathy by downregulating lipid peroxidation and protein carbonylation.^9^ Furthermore, syringic acid mitigates nitric oxide synthase inhibitor‐induced hypertension by alleviating oxidative stress.^10^ Gallic acid, gentisic acid, protocatechuic acid, syringic acid and vanillic acid belong to the class of benzoic acids.^11^ Recently, we had reported that gallic acid, gentisic acid or protocatechuic acid alleviated cardiac hypertrophy and fibrosis in the isoproterenol or pressure overload‐induced hypertrophy mouse models.^12^, ^13^, ^14^ Several phytochemicals, including apigenin, baicalein, berberine hydrochloride and emodin, suppress stress‐induced hypertrophy.^15^ However, the therapeutic effects of syringic acid on cardiac hypertrophy have not been elucidated.

In this study, the therapeutic effects of syringic acid on isoproterenol‐induced cardiac hypertrophy and fibrosis and the underlying mechanisms were examined. Syringic acid exerted preventive effects on cardiac hypertrophy and fibrosis by downregulating Ereg.

## 2 MATERIALS AND METHODS

### 2.1 Reagents

Syringic acid (4‐hydroxy‐3,5‐dimethoxybenzoic acid; cat no. S6881) and isoproterenol (cat no. I5627) were obtained from Sigma‐Aldrich Co. (St. Louis, MO, USA). Anti‐Actb (cat no. sc‐47,778), anti‐Fn1 (cat no. sc‐59,826), anti‐Col1a1 (cat no. sc‐293,182) and anti‐Nppb (cat no. sc‐271,185) antibodies were purchased from Santa Cruz Biotechnology (Dallas, TX, USA). Alexa Fluor 488 phalloidin (cat no. A12379) was obtained from Invitrogen (Eugene, OR, USA).

### 2.2 Animal model of cardiac hypertrophy

All animal procedures were approved by the Animal Experimental Committee of Chonnam National University Medical School (CNUH IACUC‐21042) and performed according to the Guide for the Care and Use of Laboratory Animals (US National Institutes of Health Publications, 8th edition, 2011).

To induce cardiac hypertrophy, male CD‐1 mice aged 8 weeks with an average bodyweight of approximately 33 g were infused with isoproterenol (25 mg/kg bodyweight/day) using an osmotic minipump (Alzet). Mice were anaesthetised by intraperitoneally injecting ketamine (120 mg/kg bodyweight) and xylazine (6.2 mg/kg bodyweight). The animals were randomly divided into the following three groups (*n* = 8/group): vehicle‐treated sham, isoproterenol‐treated and isoproterenol/syringic acid (100 mg/kg bodyweight/day)‐treated groups. Isoproterenol was solubilized in 0.1% ascorbic acid and 0.9% physiological saline, while syringic acid was dissolved in dimethyl sulfoxide (DMSO) and diluted using 0.9% physiological saline.

To determine the preventive effect of syringic acid, mice were pretreated with vehicle (DMSO) or syringic acid (100 mg/kg bodyweight/day; total volume = 400 μl) 7 days before isoproterenol infusion. Next, the animals were co‐treated with isoproterenol and syringic acid for 5 days (*n* = 8 per group).

### 2.3 Echocardiography

The echocardiography procedure was performed using the Vivid S5 system model with a 13‐MHz linear array transducer (GE Healthcare, Chicago, USA). M‐mode images and recordings were obtained from the parasternal short‐axis view of the mouse left ventricle at the level of the papillary muscle. Left ventricular posterior and interventricular septal thicknesses were measured from the images, while left ventricular end‐diastolic diameter and left ventricular end‐systolic diameter were measured from M‐mode recordings.

### 2.4 Histological analysis and Picrosirius red staining

Mice were sacrificed by exposing them to carbon dioxide for 2–3 min. The cardiac tissues were soaked in 3.7% paraformaldehyde and embedded in paraffin. The paraffin‐embedded tissues were sectioned to a thickness of 3 μm.

To measure the cross‐sectional area, the tissue sections were stained with haematoxylin and eosin (H&E) as previously described.^12^ The cross‐sectional area was quantified using NIS Elements software (Nikon Eclipse 80*i* microscope, Tokyo, Japan). Cardiac fibrosis was determined using Picrosirius red staining (Abcam, Cambridge, UK). The rehydrated cardiac tissue sections were completely covered with Picrosirius red solution for 1 h. The samples were rapidly rinsed twice with 0.5% acetic acid solution, followed by rinsing with absolute alcohol for 1 min. Next, the samples were cleared with xylene and mounted using Canada balsam. Digital images were captured under a microscope (Nikon, Japan).

### 2.5 Quantitative real‐time polymerase chain reaction (qRT‐PCR)

Total RNA was extracted from the cardiac tissues using TRIzol reagent (Invitrogen, Carlsbad, CA, USA). The isolated RNA (1 μg) was reverse‐transcribed into complementary DNA (cDNA) using TOPscript RT DryMIX (Enzynomics, Daejeon, South Korea). The PCR primers used in this study are shown in Table 1. qRT‐PCR analysis was performed using a 7500 Real‐Time PCR system with the SYBR Green PCR kit (Enzynomics). The expression levels of the target gene were normalized to those of *Gapdh* (housekeeping gene).

**TABLE 1:** List of primers used in quantitative real‐time polymerase chain reaction analysis

Genes	Primer sequences (5′ to 3′)
*Gapdh* (rat)	F: AACCCATCACCATCTTCCAGGAGC R: ATGGACTGTGGTCATGAGCCCTTC
*Nppa* (rat)	F: GCTCGAGCAGATCGCAAAAG R: GAGTGGGAGAGGTAAGGCCT
*Nppb* (rat)	F: GACGGGCTGAGGTTGTTTTA R: ACTGTGGCAAGTTTGTGCTG
*Col1a1* (rat)	F: ACCCCAAGGAGAAGAAGCAT R: AGGTTGCCAGTCTGTTGGTC
*Ereg* (rat)	F: CACCGAGAGAAGGATGGAGA R: GTGTCCATGCAAGCAGTAGC
*Ngfr* (rat)	F: GAGCGGACTGAGCTAGAAGC R: GTTCACACACGGTCTGGTTG
*Myc* (rat)	F: ACGGCCTTCTCTTCTTCCTC R: GGTTGCCTCTTTTCCACAGA
*Fn1* (rat)	F: AGCAAATCGTGCAGCCTCCG R: CCCCCTTCATGGCAGCGATT
*Gapdh* (mouse)	F: GCATGGCCTTCCGTGTTCCT R: CCCTGTTGCTGTAGCCGTATTCAT
*Nppa* (mouse)	F: TGGAGGAGAAGATGCCGGTAGAAGAT R: AGCGAGCAGAGCCCTCAGTTTGCT
*Nppb* (mouse)	F: CTGAAGGTGCTGTCCCAGAT R: GTTCTTTTGTGAGGCCTTGG
*Col1a1* (mouse)	F: GAGCGGAGAGTACTGGATCG R: GCTTCTTTTCCTTGGGGTTC
*Fn1* (mouse)	F: GATGCACCGATTGTCAACAG R: TGATCAGCATGGACCACTTC
*Ereg* (mouse)	F: CGCTGCTTTGTCTAGGTTCC R: CAGTAGCCGTCCATGTCAGA
*Ngfr* (mouse)	F: CAACCAGACCGTGTGTGAAC R: CCAGTCTCCTCGTCCTGGTA
*Myc* (mouse)	F: CAACGTCTTGGAACGTCAGA R: TCGTCTGCTTGAATGGACAG

### 2.6 Western blotting

Total protein was extracted from the cardiac tissues using radioimmunoprecipitation assay lysis buffer (150 mM NaCl, 1% Triton X‐100, 1% sodium deoxycholate, 50 mM Tris–HCl [pH 7.5], 2 mM ethylenediaminetetraacetic acid, 1 mM phenylmethylsulfonyl fluoride, 1 mM dithiothreitol, 1 mM Na_3_VO_4_ and 5 mM NaF) supplemented with a protease inhibitor cocktail (Calbiochem/EMD Millipore, Billerica, MA, USA). Proteins (40 μg) were resolved using sodium dodecyl sulphate‐polyacrylamide gel electrophoresis. The resolved proteins were transferred to a polyvinylidene difluoride membrane (0.45 μm). The membrane was blocked with 5% skim milk in Tris‐buffered saline containing Tween 20 buffer (20 mM Tris, 200 mM NaCl and 0.04% Tween 20) for 1 h at 25°C. Next, the membrane was incubated with primary antibodies overnight at 4°C, followed by incubation with anti‐rabbit or anti‐mouse horseradish peroxidase‐conjugated secondary antibodies (1:5000) for 1 h at 25°C. Immunoreactive signals were developed using Immobilon Western blotting detection reagents (EMD Millipore, Billerica, MA, USA). The protein band intensity was quantified using ImageJ software (https://imagej.net/).

### 2.7 Cell viability

H9c2 cells cultured in 24‐well plates were incubated with 1, 10 or 100 μM syringic acid for 24 h. Next, the cells were incubated with 200 μl of 10% foetal bovine serum (FBS) and 50 μl of 3‐(4,5‐dimethylthiazol‐2‐yl)‐2,5‐diphenyltetrazolium bromide solution for 2 h. The reagents were aspirated, and the cells were incubated with DMSO for 10 min. The absorbance of the mixture at 570 nm was measured.

### 2.8 Cell culture and cell size measurement

H9c2 cells were cultured in Dulbecco's modified Eagle's medium supplemented with 10% FBS at 5% CO_2_ and 37°C in an incubator. To determine cell size, cells were seeded on coverslips at a density of 1 × 10^4^ cells/well, serum‐starved overnight, and pretreated with vehicle or syringic acid (10 μM) 3 h before isoproterenol stimulation. Next, the cells were co‐treated with isoproterenol (10 μM) and syringic acid for 24 h. Cells were fixed with 3.7% paraformaldehyde for 30 min, washed twice with phosphate‐buffered saline, permeabilized with 0.2% Triton X‐100 and incubated with Alexa Fluor 488 phalloidin (1:200) for 45 min. The nuclei were stained with 4′,6‐diamidino‐2‐phenylindole, and the samples were sealed with a glass slide. The cell size was measured using NIS Elements Software Version AR 3.0 (https://www.nikonmetrology.com/images/brochures/nis‐elements‐en.pdf, Nikon, Tokyo, Japan).

### 2.9 RNA sequencing

Total RNA concentration was determined using Quant‐IT RiboGreen (#R11490; Invitrogen). The integrity of the total RNA was examined using the TapeStation RNA Screentape (#5067–5576; Agilent, Santa Clara, CA, USA). Only high‐quality RNA preparations (RNA integrity number > 7.0) were used for library construction.

A library was prepared with 0.5 μg of total RNA using the Illumina TruSeq stranded total RNA library prep gold kit (#20020599; Illumina, Inc., San Diego, CA, USA). The rRNA in the sample was removed using the Ribo‐Zero rRNA removal kit (Human/Mouse/Rat Gold) (Illumina, Inc). Next, the RNAs were fragmented into small pieces using divalent cations under elevated temperatures. The cleaved RNA fragments were reverse‐transcribed into first‐strand cDNA using SuperScript II reverse transcriptase (#18064014; Invitrogen) and random primers. Next, second‐strand cDNA synthesis was performed using DNA polymerase I, RNase H and dUTP. The cDNA fragments were subjected to end repair, single adenine base addition and adapter ligation. The products were purified and PCR‐amplified to obtain the final cDNA library.

The libraries were quantified using the KAPA library quantification kits for Illumina sequencing platforms according to the qPCR Quantification Protocol Guide (#KK4854; KAPA BIOSYSTEMS) and qualified using the TapeStation D1000 ScreenTape (# 5067–5582; Agilent Technologies). Indexed libraries were then sequenced using Illumina NovaSeq (Illumina, Inc.). Paired‐end (2 × 100 bp) sequencing was performed at Macrogen Inc.

### 2.10 Transfection

To knockdown *Ereg*, H9c2 cells were transfected with control short‐interfering RNA (siRNA) or siRNA against *Ereg* (si‐Ereg; 100 nM, Bioneer, Daejeon, South Korea) using RNAiMAX reagent for 24 h, following the manufacturer's instructions. The transfected cells were serum‐starved overnight and treated with isoproterenol for 9 h.

### 2.11 Statistical analysis

All statistical analyses were performed using GraphPad Prism 8 (GraphPad Software, La Jolla, CA, USA) and IBM‐SPSS Statistics for Windows software (ver. 25.0; SPSS Inc., Chicago, IL, USA). The data are expressed as mean ± standard error of mean. The means between two groups were compared using the Student's t‐test, whereas those between three or more groups were compared using one‐way analysis of variance, followed by Bonferroni *post hoc* test.

## 3 RESULTS

### 3.1 Syringic acid pretreatment alleviates cardiomyocyte hypertrophy and downregulates the expression of cardiac hypertrophy and fibrosis‐related genes

**FIGURE 1:**
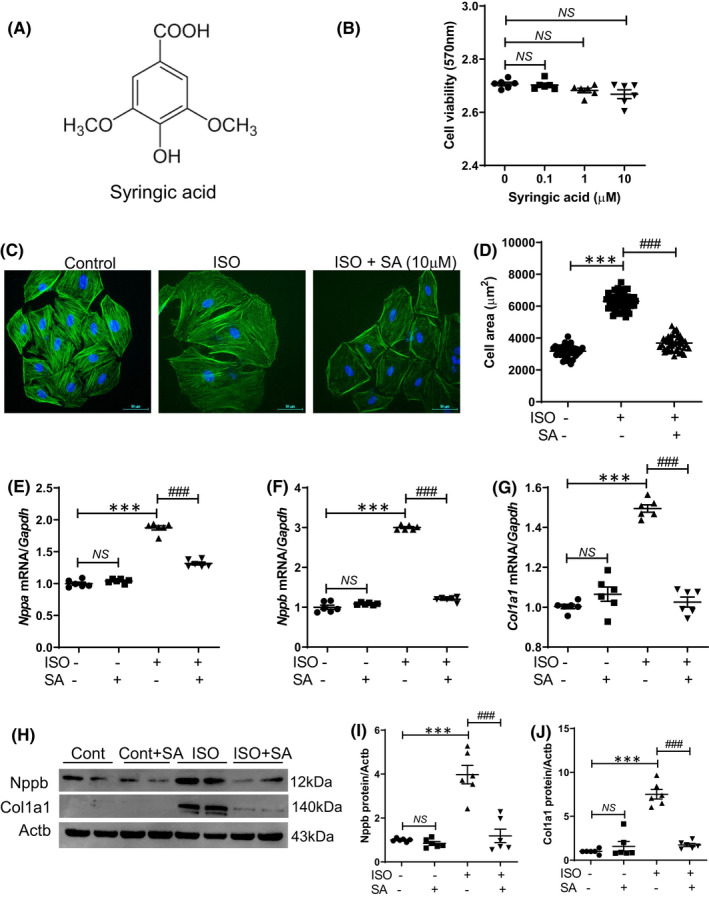
Syringic acid pretreatment mitigates isoproterenol‐induced upregulation of cardiac hypertrophy and fibrosis marker genes in vitro. (A) Chemical structure of syringic acid. (B) The viability of H9c2 cells treated with three different concentrations of syringic acid for 24 h was evaluated (*n* = 6). NS, not significant. (C, D) H9c2 cells were serum‐starved overnight and pretreated with vehicle or syringic acid (10 μM) for 1 h, followed by co‐treatment with isoproterenol (ISO, 10 μM) for 24 h. Alexa 488 phalloidin staining was performed to determine cell size. Representative images of cells (C) and quantification of cell size (D) (*n* = 40). Scale bar = 50 μm. *** p < 0.001; ^*###*^ *p <* 0.001. (E–J) H9c2 cells were pretreated with vehicle or syringic acid (10 μM) for 1 h before isoproterenol (10 μM for 9 h) stimulation. mRNA expression levels of *Nppa* (E), *Nppb* (F) and *Col1a1* (G) were determined using quantitative real‐time polymerase chain reaction (*n* = 6). The protein expression levels (H–J) were quantified using Western blotting analysis. *** p < 0.001; ^*###*^ *p <* 0.001; ^*@@@*^ *p <* 0.001; NS, not significant. Data are presented as mean ± standard error of mean. Statistics: one‐way analysis of variance, followed by Bonferroni post hoc test. ISO, isoproterenol; SA, syringic acid

Syringic acid (4‐hydroxy‐2,3‐dimethoxybenzoic acid), a phenolic compound, is found in fruits and vegetables (Figure 1A). In this study, the effects of syringic acid on cardiomyocyte hypertrophy were examined. Syringic acid was not cytotoxic to H9c2 cells up to a concentration of 10 μM (Figure 1B). Alexa Fluor 488 phalloidin staining revealed that pretreatment with syringic acid markedly mitigated the isoproterenol‐induced enhanced cardiomyocyte size (Figure 1C,D). Next, the effects of syringic acid on the expression levels of cardiac hypertrophy and fibrosis‐related marker genes were examined. Pretreatment with syringic acid markedly mitigated the isoproterenol‐induced upregulation of *Nppa*, *Nppb* and *Col1a1* mRNA levels in H9c2 cells (Figure 1E–G). The results of Western blotting analysis were consistent with those of qRT‐PCR analysis (Figure 1H–J).

### 3.2 Syringic acid alleviates cardiac hypertrophy in isoproterenol‐treated mice

**FIGURE 2:**
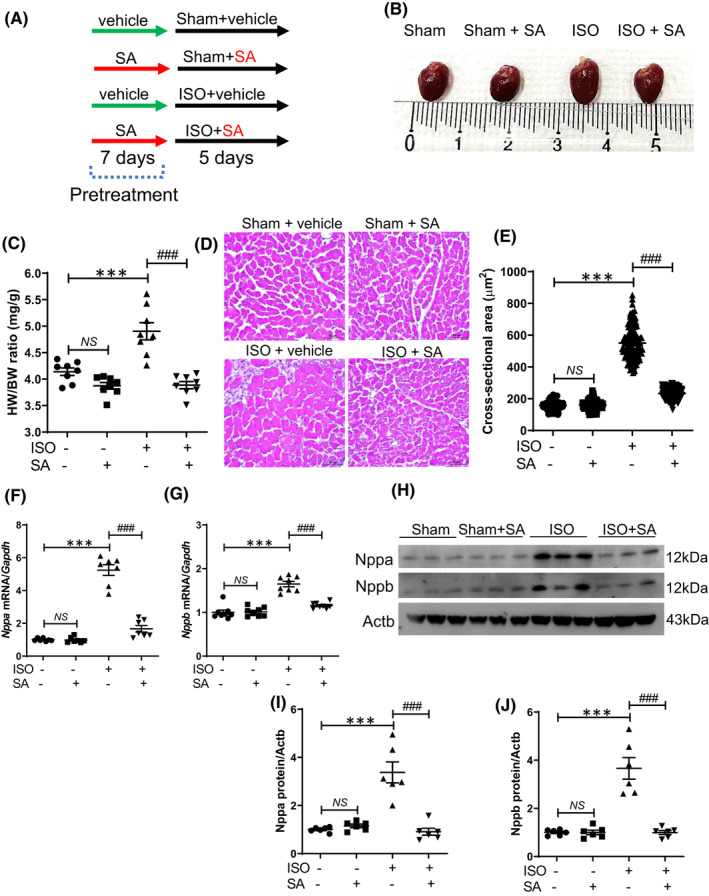
Syringic acid alleviates cardiac hypertrophy in isoproterenol‐treated mice. (A) Schematic representation of the experimental protocol. Syringic acid administration was initiated 7 days before isoproterenol infusion (5 days) in mice (sham + vehicle (dimethyl sulfoxide (DMSO), *n* = 8), sham + syringic acid (SA, 100 mg/kg bodyweight/day, *n* = 8), ISO + vehicle (DMSO, *n* = 8), ISO + SA (100 mg/kg bodyweight/day, *n* = 8)). (B) Representative images of whole hearts. (C) Heart weight to bodyweight (HW/BW) ratio (*n* = 8). *** p < 0.001; ^*###*^ *p <* 0.001; NS, not significant. (D) Representative images of haematoxylin and eosin (H&E)‐stained sections. Scale bar = 50 μm. (E) Quantification of cross‐sectional area of the cardiac tissues. *** p < 0.001; ^*###*^ *p <* 0.001; NS, not significant. (F, G) *Nppa* and *Nppb* mRNA levels were examined using quantitative real‐time polymerase chain reaction and normalized to those of *Gapdh*. * p < 0.05; *** p < 0.001; ^*###*^ *p <* 0.001; NS, not significant. (H) Nppa and Nppb levels in the cardiac tissues. Actb was used as a loading control. (I, J) Quantification of Nppa and Nppb protein levels (*n* = 6). *** p < 0.001; ^*###*^ *p <* 0.001

A mouse model of cardiac hypertrophy was pretreated with syringic acid for 7 days and co‐treated with syringic acid and isoproterenol for 5 days (Figure 2A). Pretreatment with syringic acid decreased the heart weight to bodyweight ratio in the isoproterenol‐treated group but not in the sham group (Figure 2B,C). H&E staining was performed to measure the mouse cardiomyocyte size and the area of individual cells. Syringic acid mitigated the isoproterenol‐induced enhanced cardiomyocyte cross‐sectional area (Figure 2D,E). Next, the expression levels of cardiac hypertrophic markers were examined using qRT‐PCR and Western blotting analyses. Syringic acid mitigated the isoproterenol‐induced upregulation of Nppa and Nppb mRNA and protein levels (Figure 2F,J).

### 3.3 Pretreatment with syringic acid mitigates pathological cardiac remodelling in isoproterenol‐treated mice

**FIGURE 3:**
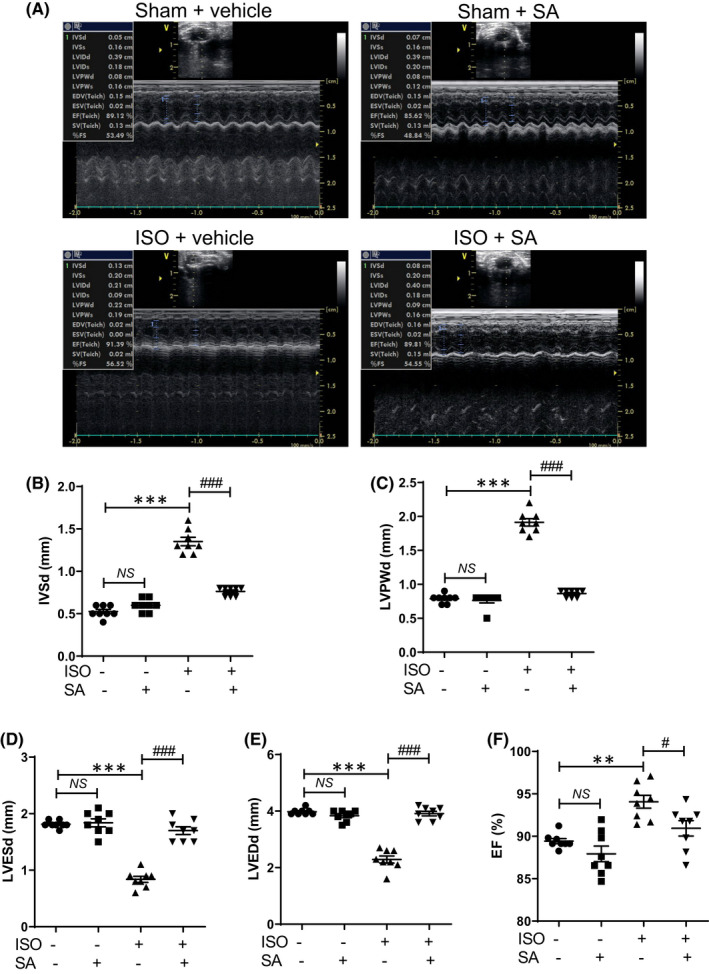
Syringic acid pretreatment mitigates isoproterenol‐induced pathological cardiac remodelling in mice. (A) Syringic acid administration was started 7 days before isoproterenol infusion (5 days) in mice (sham + vehicle (*n* = 8), sham + SA (*n* = 8), ISO + vehicle (*n* = 8) and ISO + SA (*n* = 8)). Representative M‐mode echocardiograms. (B, C) Quantification of the thickness of the interventricular septum (IVSd) and left ventricular posterior wall (LVPWd) (*n* = 8 per group). *** p < 0.001; ^*###*^ *p <* 0.001; NS, not significant. (D, E) Quantification of left ventricular end‐systolic diameter (LVESd) and left ventricular end‐diastolic diameter (LVEDd) (*n* = 8 per group). *** p < 0.001; ^*###*^ *p <* 0.001; NS, not significant. (F) Ejection fraction (%). ** p < 0.01; ^*#*^ *p <* 0.05; NS, not significant. Data are presented as mean ± standard error of mean. Statistics: one‐way analysis of variance, followed by Bonferroni *post hoc* tests. ISO, isoproterenol; SA, syringic acid

Next, echocardiography was performed to further elucidate the effects of syringic acid on cardiac hypertrophy. Syringic acid pretreatment mitigated the isoproterenol‐induced enhanced interventricular septum thickness and left ventricular posterior wall thickness (Figure 3A–C). Additionally, syringic acid markedly mitigated the isoproterenol‐induced decreased left ventricular internal dimension at the end of systole and diastole (Figure 3D–E). The ejection fraction in the isoproterenol group was higher than that in the sham group (Figure 3F).

### 3.4 Syringic acid alleviates cardiac fibrosis in isoproterenol‐treated mice

**FIGURE 4:**
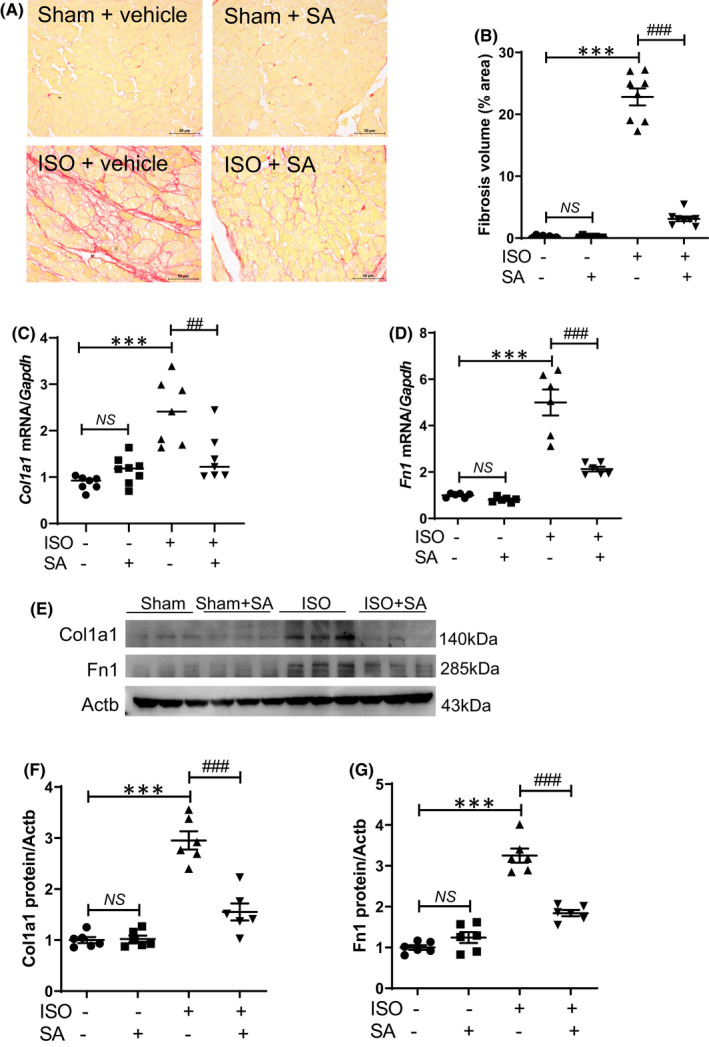
Syringic acid pretreatment mitigates isoproterenol‐induced cardiac fibrosis in mice. (A, B) Syringic acid administration was started 7 days before isoproterenol infusion (5 days) in mice (sham + vehicle (*n* = 8), sham + SA (*n* = 8), ISO + vehicle (*n* = 8) and ISO + SA (*n* = 8)). Representative images (A) of Picrosirius red‐stained cardiac tissues and quantification of staining intensity (B). Scale bar = 50 μm. *** p < 0.001; ^*###*^ *p <* 0.001. mRNA levels of *Col1a1* (C) and *Fn1* (D) were determined using quantitative real‐time polymerase chain reaction and normalized to those of *Gapdh*. **p < 0.01 and *** p < 0.001; ^*#*^ *p <* 0.05 and ^*###*^ *p <* 0.001. (E–G) Representative images of cardiac Col1a1 and Fn1 levels (*n* = 6 per group) and their quantification. Actb was used as a loading control. ISO and SA indicate isoproterenol and syringic acid, respectively. Data are presented as mean ± standard error of mean. Statistics: one‐way analysis of variance, followed by Bonferroni *post hoc* test

To evaluate the preventive effect of syringic acid on cardiac fibrosis, Picrosirius red staining, qRT‐PCR and Western blotting analyses were performed. Pretreatment with syringic acid markedly mitigated the isoproterenol‐induced upregulation of collagen deposition (pink) in the interstitial cardiomyocytes (Figure 4A,B). The mRNA and protein levels of Col1a1 and Fn1 in the isoproterenol‐treated group were higher than those in the sham group. However, syringic acid mitigated the isoproterenol‐induced upregulation of Col1a1 and Fn1 mRNA and protein levels (Figure 4C,D). Western blotting analysis revealed that syringic acid pretreatment mitigated the isoproterenol‐induced upregulation of cardiac Col1a1 and Fn1 levels (Figure 4E–G).

### 3.5 Syringic acid mitigates isoproterenol‐induced upregulation of cardiac Ereg levels

**FIGURE 5:**
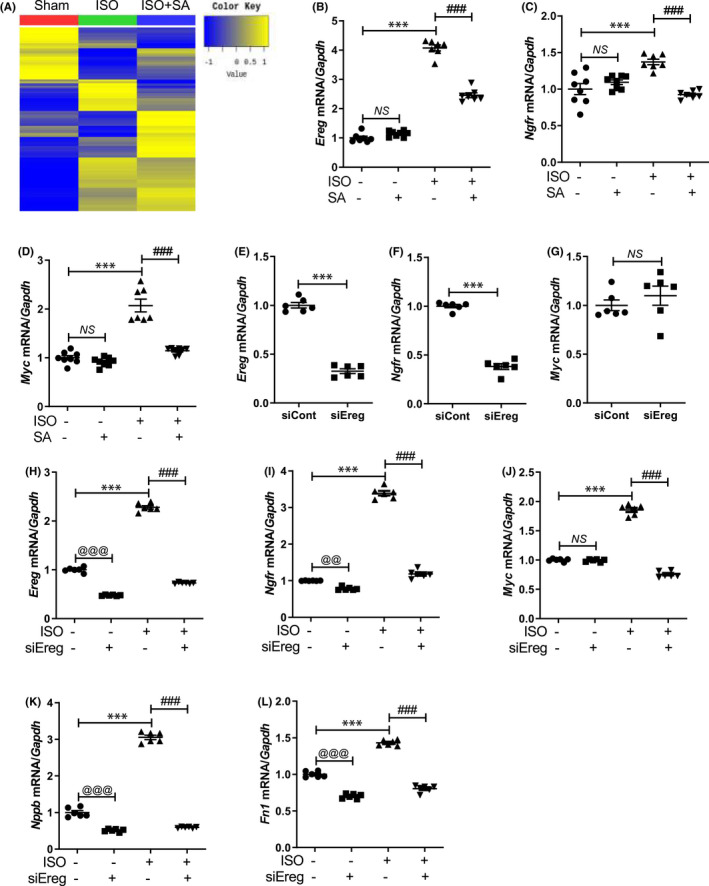
Syringic acid downregulates the expression levels of *Ereg*, *Ngfr* and *Myc*. (A) Heatmap of differentially expressed genes in the cardiac tissues of the sham + vehicle (*n* = 8), ISO + vehicle (*n* = 8) and ISO + SA (*n* = 8) groups. (B–D) RNA sequencing results of MAPK signalling‐related genes were validated using quantitative real‐time polymerase chain reaction. (E–G) *Ereg*, *Ngfr* and *Myc* mRNA levels in H9c2 cells transfected with si‐control or si‐Ereg. (H–L) *Ereg*, *Ngfr*, *Myc*, *Nppb* and *Fn1* mRNA levels in H9c2 cells transfected with si‐control or si‐Ereg and treated with isoproterenol

To investigate the syringic acid‐regulated genes, RNA‐seq was performed. Several MAPK signalling‐related genes, which were enriched in the Kyoto Encyclopedia of Genes and Genomes pathway analysis, were examined. Heatmap analysis of differentially expressed genes revealed that the cardiac mRNA levels of *Ereg*, *Ngfr* and *Myc* in the isoproterenol‐treated group were upregulated when compared with those in the sham group. However, syringic acid mitigated the isoproterenol‐induced upregulation of cardiac *Ereg*, *Ngfr* and *Myc* mRNA levels (Figure 5A). The upregulation of these three genes was confirmed using qRT‐PCR (Figure 5B–D).

siRNA transfection was performed to investigate the role of Ereg. Transfection with si‐Ereg significantly downregulated the endogenous *Ereg* mRNA levels in H9c2 cells (Figure 5E). Additionally, transfection with si‐Ereg downregulated the mRNA levels of *Ngfr* but not those of *Myc* (Figure 5F,G). Next, the effect of *Ereg* knockdown on isoproterenol‐treated H9c2 cells was examined. Transfection with si‐Ereg mitigated the isoproterenol‐induced upregulation of Ereg, Ngfr and Myc (Figure 5H–J). Additionally, si‐Ereg transfection downregulated the *Nppb* and *Fn1* mRNA levels with or without isoproterenol stimulation (Figure 5K,L).

### 3.6 *Ereg* knockdown mitigated isoproterenol‐induced cardiomyocyte hypertrophy and downregulated fibronectin expression

**FIGURE 6:**
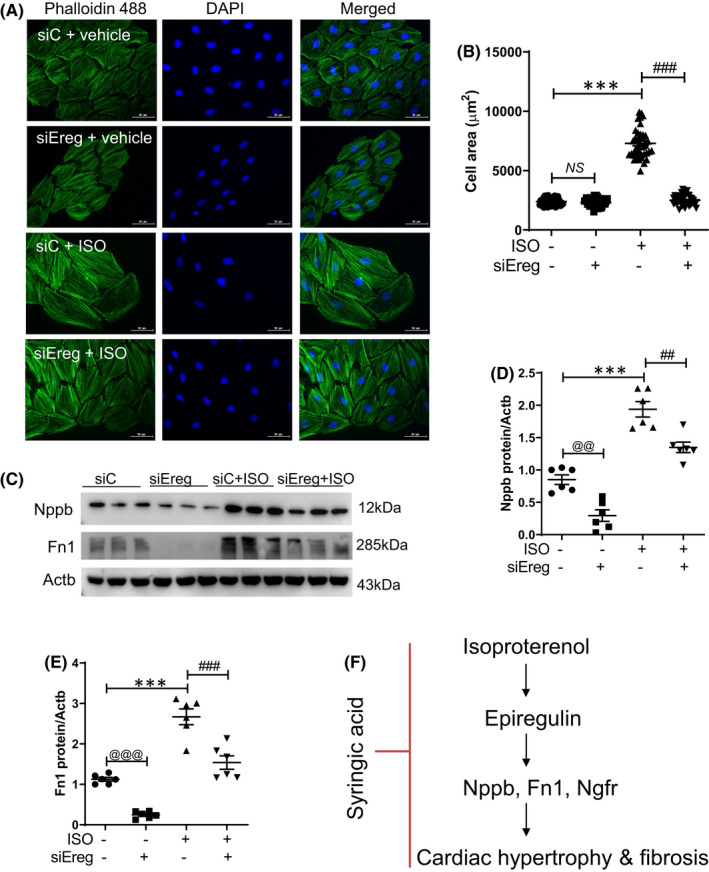
*Ereg* knockdown mitigated isoproterenol‐induced cardiomyocyte hypertrophy and fibronectin (A) Representative images of phalloidin 488‐stained cells. Green and blue indicate phalloidin 488‐stained cells and nucleus, respectively. Scale bar = 50 μm. (B) Cell area of si‐Ereg‐transfected and isoproterenol‐stimulated H9c2 cells. *** p < 0.001; ^*###*^ *p <* 0.001. (C) Representative Western blotting images of si‐Ereg‐transfected and isoproterenol‐treated cells. (D, E) Quantification of Nppb and Fn1 protein levels. *** p < 0.001; ^*##*^ *p <* 0.01 and ^*###*^ *p <* 0.001; ^*@@*^ *p <* 0.01 and ^*@@@*^ *p <* 0.001. (F) Schematic diagram of the mechanism of syringic acid involved in the suppression of cardiac hypertrophy and fibrosis. Isoproterenol upregulates *Ereg* mRNA levels in vivo and in vitro. *Ereg* knockdown mitigates the isoproterenol‐induced upregulation of Nppb, Fn1 and Ngfr. Syringic acid alleviates cardiac hypertrophy and fibrosis. Nppb, natriuretic peptide; Fn1, fibronectin; Ngfr, nerve growth factor receptor

To examine the effect of Ereg on cardiomyocyte size, cell area was measured after F‐actin staining. Cell size was similar between si‐Ereg‐transfected and si‐control‐transfected cells in the absence of isoproterenol stimulation (Figure 6A,B). In the si‐control‐transfected cells, the cell area in the isoproterenol‐treated group was significantly higher than that in the vehicle‐treated group. The mitigation of isoproterenol‐induced increased cell size in the si‐Ereg‐transfected group was higher than that in the si‐control‐transfected group (Figure 6A,B). Western blot analysis revealed that Nppb levels in si‐Ereg‐transfected cells were significantly downregulated compared with those in the si‐control‐transfected cells (Figure 6C,D). Transfection with si‐Ereg mitigated the isoproterenol‐induced upregulation of Nppb levels. Fn1 levels were markedly downregulated in si‐Ereg‐transfected cells (Figure 6C,E). Transfection with si‐Ereg significantly mitigated the isoproterenol‐induced upregulation of Fn1 levels.

## 4 DISCUSSION

This study demonstrated that syringic acid mitigates isoproterenol‐induced cardiac hypertrophy and fibrosis by downregulating Ereg (Figure 6F). RNA‐seq analysis revealed that *Ereg*, *Myc* and *Ngfr* mRNA levels in isoproterenol‐treated mice were upregulated compared with those in the sham group. Pretreatment with syringic acid mitigated the isoproterenol‐induced upregulation of *Ereg*, *Myc* and *Ngfr* mRNA levels. RNA‐seq data were validated using qRT‐PCR. The results of siRNA transfection experiments revealed that Ereg modulates Ngfr but not Myc. However, *Ereg* knockdown mitigated the isoproterenol‐induced upregulation of Ngfr and Myc levels. Previous studies have reported that Ereg expression is upregulated in the rat acute myocardial infarction model. However, one study reported that EREG silencing promotes left ventricular remodelling and increases infarct size.^16^ The reasons for these conflicting results are not known. *EREG* mRNA levels in human atherosclerotic arteries were upregulated when compared with those in healthy arteries. Additionally, the downstream effectors of EREG (ERK and p38 MAPK signalling) were upregulated in human atherosclerotic arteries.^17^ EREG binds to the epidermal growth factor (EGF) receptor (ErbB1, ErbB2, ErbB3 or ErbB4). This study did not evaluate the expression of EGF receptor in the cardiac hypertrophic animal model. *Ereg* knockdown directly suppressed the expression of hypertrophic (*Nppb*) and fibrotic (*Fn1*) marker genes in vitro. EREG‐regulated hypertrophy and fibrosis‐related genes are not directly related to cardiomyocyte size reduction. However, siRNA‐mediated *Ereg* knockdown almost completely mitigated the isoproterenol‐induced enhanced cell area and fibronectin expression. These findings indicate that EREG is associated with cardiac hypertrophy and fibrosis. EREG is a ligand of EGFR, which has a role in fibrosis. For example, genetic deletion of EGFR attenuated CCl_4_‐induced liver fibrosis.^18^

EREG was reported to promote the proliferation and migration of renal proximal tubular cells.^19^ Additionally, EREG promoted the migration of salivary adenoid cystic carcinoma cells by upregulating the phosphorylation of ERK, Akt, STAT3 and EGFR.^20^ The transcription factors GATA4, GATA6 and SP1 are reported to induce cardiac hypertrophy.^14^, ^21^ However, EREG does not directly increase cardiomyocyte size. The findings of this study suggest that EREG partially contributes to cardiac hypertrophy.

Pretreatment with syringic acid mitigated the isoproterenol‐induced upregulation of Ereg expression. Syringic acid is a 3,5‐dimethyl ether derivative of gallic acid. Gallic acid is reported to alleviate cardiac hypertrophy, fibrosis, cardiotoxicity, hypertension and heart failure.^12^, ^22^, ^23^, ^24^, ^25^, ^26^ We hypothesized that syringic acid exerts cardioprotective effects. Consistent with this hypothesis, syringic acid mitigated isoproterenol‐induced cardiac hypertrophy in vivo and in vitro. Additionally, syringic acid pretreatment mitigated isoproterenol‐induced enhanced heart weight and histological changes. Cardiac hypertrophic markers, such as Nppa and Nppb, were downregulated upon syringic acid treatment. Syringic acid mitigated isoproterenol‐induced pathological cardiac remodelling (narrowing of the lumen and thickening of the cardiac wall). These findings suggest that syringic acid is a potential therapeutic agent for the prevention of cardiac hypertrophy and fibrosis.

Syringic acid significantly mitigated isoproterenol‐induced cardiac fibrosis. Increased collagen deposition is related to enhanced cardiac Col1a1 expression in the cardiac hypertrophy model. The limitation of this study was that the fibrosis‐related genes were not evaluated using cardiac fibroblast cell lines.

In conclusion, syringic acid exerted cardioprotective effects by downregulating Ereg. This study, for the first time, demonstrated that downregulation of Ereg alleviates isoproterenol‐induced cardiac hypertrophy and fibrosis. Hence, syringic acid is a potential therapeutic agent for the prevention of cardiac hypertrophy and fibrosis.

## AUTHOR CONTRIBUTIONS

**Xiongyi Han:** Conceptualization (equal); data curation (equal); formal analysis (equal); investigation (equal); methodology (equal); writing – review and editing (equal). **Liyan Bai:** Conceptualization (equal); data curation (equal); formal analysis (equal); investigation (equal); methodology (equal). **Hae Jin Kee:** Conceptualization (lead); funding acquisition (equal); investigation (equal); supervision (lead); writing – original draft (lead); writing – review and editing (equal). **Myung Ho Jeong:** Funding acquisition (equal); resources (equal); supervision (supporting); writing – review and editing (equal).

## CONFLICT OF INTEREST

None of the authors have any conflicts of interest to declare.

## Data Availability

The data used to support the findings of this research are available from the corresponding author upon request.
